# Editorial Comment: Cardiovascular Morbidity in a Randomized Trial Comparing GnRH Agonist and GnRH Antagonist among Patients with Advanced Prostate Cancer and Preexisting Cardiovascular Disease

**DOI:** 10.1590/S1677-5538.IBJU.2020.05.09

**Published:** 2020-07-31

**Authors:** Felipe Lott

**Affiliations:** 1 Instituto Nacional do Câncer Rio de Janeiro RJ Brasil Instituto Nacional do Câncer - INCA, Rio de Janeiro, RJ, Brasil

Margel D ^1^^,^^2^, Peer A ^3^, Ber Y ^1^, Shavit-Grievink L ^1^^,^^4^, Tabachnik T ^1^, Sela S ^1^, et al.

## COMMENT

Retrospective studies have shown an association between androgen deprivation therapy (ADT) and an increase risk of cardiovascular disease (CVD) (1,2).

This paper is the first randomized study (phase II) on cardiovascular morbidity with high risk or metastatic prostate cancer patients with previous CVD events (CVDes) (37% experienced a myocardial infarction within a year before randomization) and treated with GnRh agonist (with 2 times more diabetes patients) or GnRh antagonist by one year period time.

The primary endpoint was to compare endothelial function using the EndoPAT 2000 device (3) that appears to predict cardiovascular outcomes (4). The secondary endpoint was CVDes.

There was no difference in the primary endpoint between the 2 groups but it occurred in the secondary one (more subject to statistical error) with 3% of major cardiovascular and cerebrovascular event in the GnRh antagonist group and 20% in the GnRh agonist one (p=0.013).

Maybe it is not appropriate to compare arms of a phase II trial especially in this small one. A large phase III trial (PRONOUNCE study) may define it better but, until now, an alert has been sent in patients with preexisting CVD (especially with a new event in the last 12 months).
